# Music Therapy in Depression: Exploring Mechanisms and Efficacy in Rat Models

**DOI:** 10.3390/brainsci15040338

**Published:** 2025-03-25

**Authors:** Jingqi Le, Wangyan Deng, Tao Le

**Affiliations:** 1 School of General Education and International Studie, Chongqing Polytechnic University of Electronic Technology, Chongqing 401331, China; lejingqi@cqcet.edu.cn (J.L.); dengwangyan@cqcet.edu.cn (W.D.); 2 College of Life Sciences, Chongqing Normal University, Chongqing 401331, China

**Keywords:** depression, music therapy, rat model, music, behavioral tests

## Abstract

**Background/Objectives**: Depression is a common mental disorder, and traditional treatments are often associated with side effects, making it particularly important to identify safe and effective alternative therapies. As a non-invasive intervention, music therapy has attracted increasing attention in the field of mental health in recent years. **Methods**: This study aimed to explore the effectiveness of music interventions in alleviating depressive symptoms through a systematic review of their effects on a rat model of depression. The databases PubMed, Embase, Medline, PsycINFO, Scopus, Web of Science, the Cochrane Library, Google Scholar, and Ovid MEDLINE were searched for publications dated between January 2010 and November 2024. **Results**: First, the construction methods and behavioral test approaches used in depressive rat models were introduced and analyzed. Second, the effects of music on the physiological and biochemical indexes, as well as the neural structure and function of depressed rats, were discussed. The types of music influencing depressive behaviors in rats were also summarized and discussed. Finally, current concerns and challenges in translating music therapy into clinical applications for depression were reviewed, alongside future prospects for its development. **Conclusions**: It is anticipated that this review will pave the way for both basic research and the clinical application of music therapy in the treatment of depression.

## 1. Introduction

Depression is a common and serious mental disorder that has become one of the major global health challenges. According to the World Health Organization, depression affects about 280 million people worldwide, or 3.8% of the global population [1,2]. Core symptoms of depression include persistent low mood, loss of interest in activities that were once enjoyable, changes in appetite and sleep patterns, and significant declines in thinking and decision-making skills [3]. These symptoms not only severely affect the patient’s daily life but also exacerbate physical health problems. The most severe forms of depression can lead to suicidal behavior, and around 800,000 people worldwide kill themselves each year due to depression [4]. Currently, drug therapy and psychotherapy have been the primary intervention methods. However, they often have certain limitations. Drug treatment is frequently associated with side effects, drug resistance, and addiction, resulting in poor adherence [5]. Although psychotherapy is effective, its high cost and long treatment cycle prevent many patients from committing to the long treatment. Therefore, exploring alternative treatment methods that are low-cost, highly effective, and have fewer side effects has become an important topic in modern medical research.

As a non-invasive intervention, music therapy has been widely applied in the field of mental health, demonstrating remarkable efficacy, particularly in the treatment of depression [6]. Music is believed to improve mental health by regulating mood, alleviating anxiety, promoting neuroplasticity, and improving brain function [7]. Studies have found that classical music can activate the brain’s reward system and improve emotional regulation, while soft music and natural sounds can effectively ease stress responses by reducing cortisol levels [8,9]. Compared to drugs and traditional psychotherapy, music therapy offers several advantages: it is easy to implement, cost-effective, and associated with fewer side effects [10]. Music therapy is not only well-accepted by patients but also provides a gentle treatment approach, avoiding the risk of addiction often associated with pharmacological interventions [11]. In addition, the diversity of music styles allows for personalization to meet individual patient needs, enabling the selection of music with different styles, rhythms, and melodies to achieve the best therapeutic effect.

**Figure 1:**
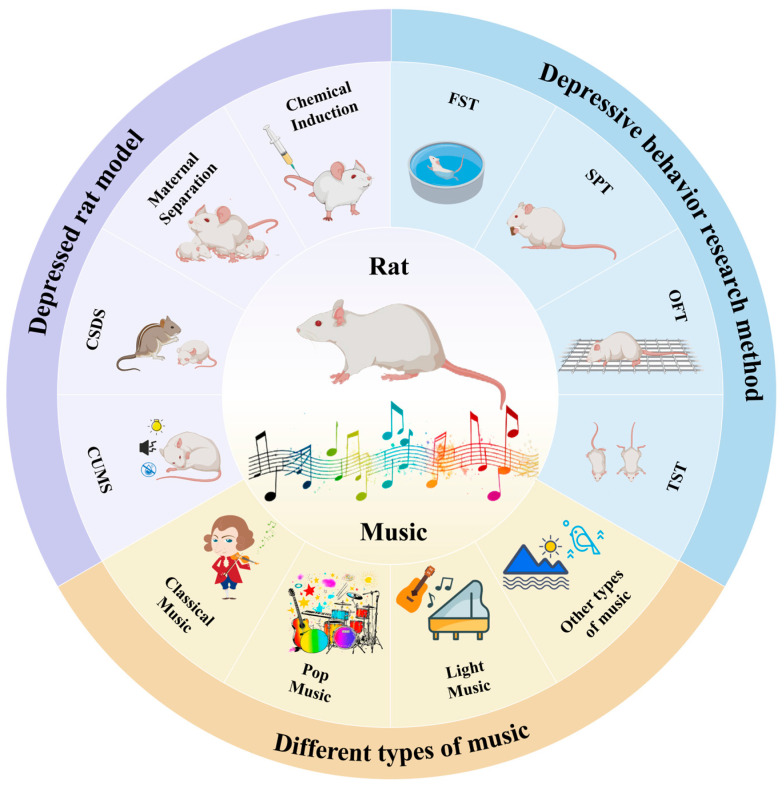
Overview of music therapy for depression based on animal models. CUMS, chronic unpredictability stress; CSDS, chronic social defeat stress; OFT, open field test; FST, forced swim test; SPT, sucrose preference test; TST, tail suspension test.

**Figure 2:**
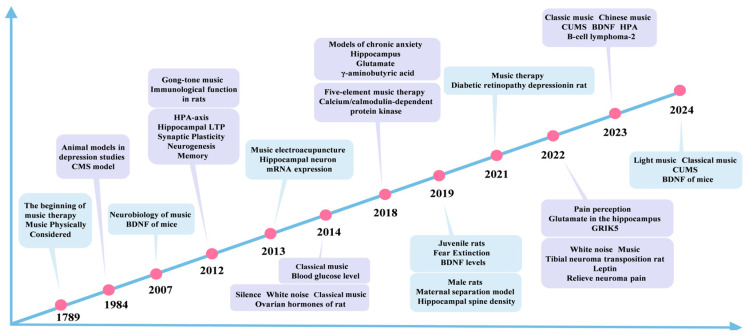
Evolution timeline of music therapy for depressed animal models based on literature analysis. CMS, chronic mild stress; BDNF, brain-derived neurotrophic factor; LTP, long-term potential; GRIK5, glutamate ionotropic receptor kainate type subunit 5; B-cell lymphoma-2.

Animal models, especially depressed rat models, have become important tools for studying the pathological mechanisms of depression and developing new therapeutic approaches. Models, such as the chronic unpredictable stress (CUMS) model and the chronic social defeat stress (CSDS) model, are commonly used to induce depression-like behaviors in rats. These models have behavioral characteristics similar to those observed in human depression, such as decreased interest, decreased activity, and weight loss [12]. Using these models, researchers have investigated the potential effects of music therapy on depression, focusing on its impact on neurotransmitters, neural circuits, and the immune system [13]. Music therapy has been shown to significantly alleviate behavioral abnormalities in depressed rats, restore social interaction and exploratory behaviors, and partially regulate the level of neurotransmitters in their brains [14]. For example, studies have found that classical and light music can effectively increase exploratory activity and exercise levels in rats, suggesting that music can mitigate depressive symptoms [15,16]. These experimental findings not only provide theoretical support for the clinical treatment of depression but also lay a solid foundation for the future application of music therapy. The purpose of this study was to systematically analyze the effects of music on the behavior of depressed rats, investigate its neurobiological mechanisms, and explore the potential of music therapy in the treatment of depression, as illustrated in Figure 1. To examine the application and evolution of music therapy, as well as the use of animal models and music types in depression treatment, this study conducted an extensive search of several databases, including PubMed, Embase, Medline, PsycINFO, Scopus, Web of Science, the Cochrane Library, Google Scholar, and Ovid MEDLINE. Searches were conducted using the terms “music” and “music therapy” and “depression” or “animal” in the search fields of title, abstract, and keywords. A total of 1312 publications were obtained. We analyzed the literature on animal models of music therapy for depression from 1789 to 2024, covering research progress on the effects of music on animal models, neurobiology, immunological function, and pain perception, as shown in Figure 2. Through a comprehensive analysis of the existing literature, this study also summarized the advantages and limitations of music therapy in alleviating depressive symptoms and provided recommendations for future research directions.

## 2. Construction of Rat Model of Depression

**Figure 3:**
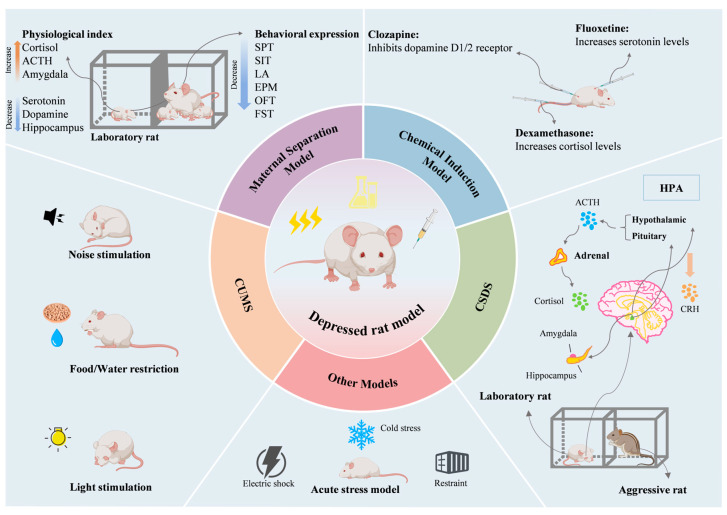
Construction of depressed rat models. ACTH, adrenocorticotropic hormone; SPT, sucrose preference test; SIT, social interaction test; LA, locomotor activity; EPM, elevated plus maze; OFT, open field test; FST, forced swim test; HPA, hypothalamic–pituitary–adrenal axis.

The rat model of depression is an important tool for studying the pathophysiological mechanisms of depression and evaluating the efficacy of new therapies (see Figure 3). These models provide an experimental basis for understanding the complexity of human depression by simulating its signs and symptoms. To explore the neurobiological mechanisms underlying depression and evaluate the effects of therapeutic interventions, it is necessary to analyze and discuss the methods used to establish various depressive rat models, their behavioral characteristics, and their applications in depression research.

### 2.1. CUMS Model

The CUMS model is one of the most common models for studying depression in rats and has been widely used to evaluate the relationship between chronic stress and depression. This model induces chronic stress responses by exposing rats to a series of low-intensity, unpredictable stressors, such as food or water restrictions, environmental changes, and noise disturbances. Under these continuous stress conditions, rats showed behavioral characteristics similar to human depression, including anhedonia (loss of pleasure), reduced activity, and other depressive-like behaviors [17,18]. The CUMS model can effectively simulate the chronic progression of depression and is especially suitable for studying the effects of chronic stress on brain function and physiological responses [17,19].

### 2.2. CSDS Model

The CSDS model induces persistent social frustration by exposing experimental rats to more aggressive rival rats. Since social stress plays an important role in many cases of human depression, the CSDS model is particularly suitable for studying depression symptoms triggered by social stress. This model has been proven to be closely correlated with the social withdrawal characteristics of human depression and effectively mimics the social avoidance behaviors commonly observed in depressed patients [20,21]. Through repeated exposure to socially frustrating environments, rats present symptoms such as the low mood and loss of social function that are similar to those seen in individuals with depression.

### 2.3. Maternal Separation Model

The maternal separation model causes long-term emotional disorders by separating newborn rats from their mothers during a critical developmental period [18]. This model is particularly useful for studying the impact of early-life stress on adult depressive symptoms, especially in exploring whether individuals exposed to early stress are more susceptible to developing depression in adulthood [22]. Research has shown that mother–infant separation has profound effects on brain development and emotional regulation in rats, increasing their risk of depression in adulthood. Therefore, this model provides a theoretical basis for investigating early interventions and preventive strategies for depression.

### 2.4. Chemically Induced Model

Chemically induced models induce depression-like behaviors in rats by administering glucocorticoids, anti-cancer drugs, or other chemicals that interfere with the neuroendocrine or immune systems [23,24]. For example, long-term injections of corticosteroids, such as dexamethasone, could mimic physiological changes commonly observed in people with depression by increasing plasma cortisol levels [25]. This experimental approach is often used to evaluate the effects of antidepressants and to analyze the effects of pharmacological interventions on depression-related neurobiological markers [26,27,28,29].

### 2.5. Other Models

In addition to the commonly used models mentioned above, other methods, such as the acute stress model and environmental change model, have also been applied in depression research [30]. These models usually induce depression-like behaviors through a single stressor or situational change. Although they have some value in certain studies, their ability to simulate long-term depressive symptoms and chronic stress responses is more limited compared to models such as CUMS and CSDS.

## 3. Behavioral Test Methods of Rat Model of Depression

### 3.1. Forced Swim Test (FST)

The FST is one of the behavioral tests commonly used to study depression, typically assessing the severity of depression by placing rats in water from which escape is impossible and recording their floating time [31]. Healthy rats tend to struggle and attempt to escape when faced with stress, while depressed rats display helpless floating and lack active responses. Studies have shown that the floating time of depressed rats in the FST is significantly prolonged, which is related to the feelings of hopelessness and negative emotions observed in human patients with depression [32].

### 3.2. Sucrose Preference Test (SPT)

The SPT is used to assess rats’ selective preference for sugar-containing solutions over water, serving as a measure of their pleasure or reward response [33]. Normal rats typically show a preference for sugar water, while depressed rats present a reduced preference, indicating a loss of pleasure (anhedonia). This phenomenon is often consistent with emotional numbness and decreased interest observed in human patients with depression [34]. The sugar-water preference experiment is one of the classic experiments for verifying the characteristic loss of pleasure in depressed rat models.

### 3.3. Open Field Test (OFT)

The OFT can assess rats’ anxiety and movement levels by measuring how much they move in an accessible open field. Healthy rats often actively explore the open field, showing higher levels of activity and staying in the central area. However, depressed rats typically show reduced activity and behaviors to avoid the central areas, suggesting the presence of anxiety or emotional suppression [35]. Studies have found that the activity level of depressed rats decreases significantly, with a tendency to stay in the marginal areas, indicating anxiety and the avoidance of new environments [36].

### 3.4. Tail Suspension Test (TST)

The TST is a commonly used experimental method to assess feelings of hopelessness in rats. In this test, rats are suspended in the air, and their immobility time is recorded to reflect their emotional response [37]. Depressed rats typically show longer periods of immobility, indicating a lack of active coping strategies and a strong sense of helplessness. This behavioral feature reflects the negative emotions and unresponsiveness to stress observed in human patients with depression [38,39].

## 4. Effects of Music on Physiological and Biochemical Indexes of Depressed Rats

### 4.1. Cortisol Levels

Changes in cortisol levels, a major stress hormone, are important indicators for assessing depressive status and the effects of interventions [40]. In both depressed patients and animal models, cortisol levels are often elevated, reflecting the activation of the stress response and the hypothalamic–pituitary–adrenal (HPA) axis [41,42,43]. Studies have shown that music intervention can significantly reduce cortisol levels in depressed rats, suggesting that music may reduce the stress response by modulating the activity of the HPA axis.

### 4.2. Neurotransmitter Levels

Neurotransmitters, such as dopamine, serotonin, and norepinephrine, play a key role in regulating mood and behavior [44]. Depression is often associated with the dysfunction of these neurotransmitters [45]. Music intervention has been found to modulate neurotransmitter levels in the brains of depressed rats. Specifically, music can increase the concentrations of dopamine and serotonin in the brain, improving depression-related behaviors [46,47]. One study measured neurotransmitter levels in rat brain tissues using high-performance liquid chromatography and found that music intervention significantly increased dopamine and serotonin levels in the hippocampus and prefrontal cortex [47].

### 4.3. Other Indexes

In addition to cortisol and neurotransmitters, music interventions have also been shown to affect other biochemical markers associated with depression in rodents. For example, music could affect the levels of inflammatory factors in rodents, such as tumor necrosis factor-α and interleukin-6, which are often elevated in depression and are correlated with the severity of the condition [48]. Music therapy has also been found to be effective in regulating heart rate and blood pressure in depressed patients [49,50]. These effects may be achieved through both psychological and physiological pathways, including the modulation of the autonomic nervous system, reduction of negative emotions, and improvement in sleep quality.

## 5. Effect of Music on Nervous Structure and Function in Depressed Rats

### 5.1. Brain Function and Adaptability

**Figure 4:**
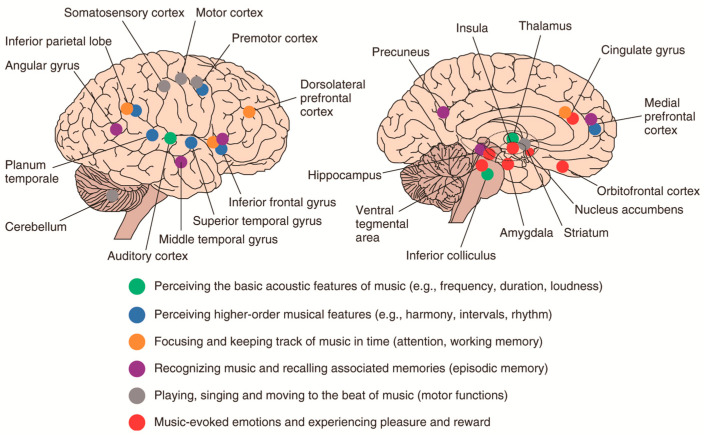
Schematic illustration of key brain areas associated with music processing-based neuroimaging studies of healthy subjects. Note that although the image displays the lateral and medial parts of the right hemisphere, many musical subfunctions are actually largely bilateral (with the exception of pitch and melody processing, which is more lateralized to the right hemisphere). Copyright © 2013 John Wiley & Sons, Ltd. [54].

The influence of music on brain function and adaptability is primarily reflected in two aspects: neural activation and neuroplasticity [51,52]. Neural activation refers to enhancing neural activity and connectivity within existing neural networks, and neuroplasticity refers to the brain’s ability to change its structure and function in response to environmental or experiential changes. Growing evidence suggests that music activates a complex and widespread bilateral network of cortical and subcortical areas that control auditory, cognitive, sensory-motor, and emotional functions in healthy subjects (see Figure 4) [53,54,55]. In a depressed rat model, music intervention was found to promote neurogenesis in the hippocampus, a region strongly associated with learning and memory [56]. For example, studies have found that rats exposed to music have increased levels of brain-derived neurotrophic factor (BDNF), neurogenesis, and neuroplasticity in the hippocampus [12,57]. However, the study was limited by its small sample size and the absence of long-term follow-up studies to assess the lasting effects of music interventions [58].

### 5.2. Neuroplasticity

**Figure 5:**
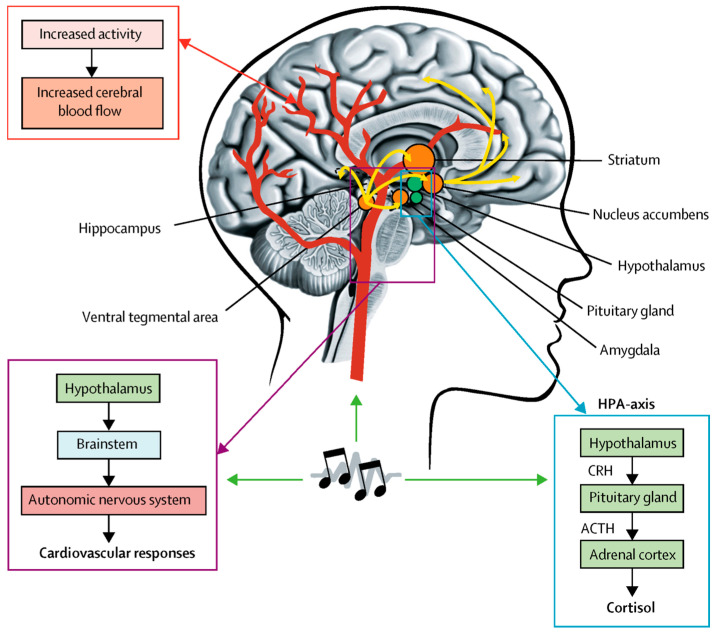
Possible neurobiological mechanisms for the rehabilitative effect of music. Orange circles and yellow arrows represent the mesolimbic system, and the green circles represent the HPA axis. ACTH = adrenocorticotropic hormone. CRH = corticotropin-releasing hormone. HPA axis = hypothalamic-pituitary–adrenal axis. (Copyright © The Lancet Neurology 2017) [53].

Functional neuroimaging studies in healthy participants have shown that music induces widespread activation across various brain networks, increasing blood flow to the medial arteries of the brain (see Figure 5). This effect is not limited to humans, and similar responses have been observed in mice. In rats, music has been shown to activate areas such as the nucleus accumbens, hippocampus, and hypothalamus, leading to increased cerebral blood flow and potentially being beneficial for recovery from neurological diseases [59]. Similar to humans, music can regulate the hypothalamic axis in rats, affecting the release of corticosteroids such as corticosterone, and this regulation has a significant effect on stress response and overall brain function [60].

It provides a favorable environment for overall recovery from neurological diseases [53]. Several studies have reported memory-related plasticity effects after listening to music, as well as neural recombination in patients with neurological disorders following music therapy [53]. Other research further supports the role of audiology-related and motion-related neuroplasticity in patients who undergo music therapy [61]. In depressed rat models, musical training has been found to increase the number and complexity of synapses in the brain, improving learning and memory retention in depressed rats [62,63]. More importantly, music therapy can increase gray matter in the frontal lobe and limbic system, leading to structural changes in these areas. While these studies provide evidence for the effects of music on neural structure and function, there are some common limitations. First, most of the relevant research has been conducted in animal models. Although we acknowledge the inherent differences between animal models and humans, the rat model has been widely accepted as an important tool for biomedical research due to its genetic, physiological, and anatomical similarities to humans. However, we also emphasize that while the rat model provides valuable insights, caution is needed when extrapolating these findings directly to humans due to species-specific differences. Second, the specific mechanism of music intervention is not fully understood, and more research is needed to explore the specific ways in which music affects neurotransmitters and neurotrophic factors.

### 5.3. BDNF

BDNF plays an important role in promoting neurogenesis and neuroplasticity through music. Studies have shown that the levels of BDNF rise after musical stimulation, which may promote the formation of new neurons and strengthen synaptic connections [60]. In rats, exposure to music has been shown to increase the expression of BDNF in the hippocampus, a key region for learning and memory, leading to improved performance on learning and memory tasks [64]. These increases in BDNF are associated with increased neuroplasticity and learning ability, providing a possible mechanism for the role of music intervention in improving cognitive function [65,66]. Research has also shown that music-induced rhythms can alleviate depressive symptoms by synchronizing theta waves in the auditory cortex with the bed nucleus of the stria terminalis and the nucleus accumbens [6]. The core finding of this study was that music with antidepressant effects could induce specific neural electrical activity in the auditory cortex, which then transmits to the deeper reward circuits in the brain, leading to the subjective enjoyment of music and subsequent antidepressant effects. Importantly, these findings are not limited to humans; similar neural mechanisms may be present in rat models. In rats, music exposure has been shown to increase BDNF expression in the hippocampus and may also increase BDNF expression in other brain regions involved in reward circuits [67,68]. Cheng et al. also explored the efficacy of music therapy in a rodent model of depression [57]. The study found that both light and classical music significantly alleviated depressive behaviors in mice, reducing corticosterone levels, increasing glucocorticoid receptor levels, and enhancing BDNF signaling, synaptic proteins, and neurogenesis. Although this study provides evidence of music therapy’s effects on BDNF, it was limited to animal models.

### 5.4. Neurotransmitters

Music can also affect the levels of neurotransmitters, such as dopamine and serotonin, which are closely linked to mood regulation, reward, and cognitive function [46,53]. Through these neurotransmitters, music may enhance the excitatory and inhibitory balance of the brain, thereby affecting the behavior and emotional state of animals [69]. A study by Hao et al. (2020) [14] explored the effects of traditional Chinese five-tone music therapy on neurotransmitters in rats. The rats were divided into a five-tone music group and a control group and exposed to music for 2 h a day for 28 days. The results showed that different types of music had different effects on glutamic acid (Glu) and gamma-aminobutyric acid (GABA) levels, for example, Gong music increased Glu and decreased GABA, while Yu music had the opposite effect. In addition, glutamine (Gln) levels were also significantly different in the Zhi, Gong, and Yu music groups. Research suggests that music therapy may affect brain function by modulating neurotransmitter levels [14].

## 6. Effects of Different Types of Music on Depressive Behavior in Rats

### 6.1. Classical Music

Classical music, especially the works of composers such as Mozart and Beethoven, has become an important type of music in depression intervention studies due to its complex melodies, harmonious rhythms, and rich emotional expression. Studies have shown that classical music can significantly improve depressive behaviors [70]. In one study, rats were subjected to a maternal separation model to simulate early life stress, and they showed reduced social skills, increased anxiety and depressive behaviors, and decreased dendritic spine density in the CA1 region of the hippocampus as adults. However, the rats were allowed to listen to Mozart’s K.448 for 12 h a day from day 21 to day 76, with the music played through standard laboratory speakers at a volume set to 60 decibels to ensure the animals could hear the music clearly while avoiding stress to the animals at too high a volume. Reversing these negative effects of maternal separation improved emotional behavior and promoted neuronal plasticity, suggesting that a music-rich environment can positively influence behavioral and neurological deficits caused by early life stress [71].

### 6.2. Light Music

Light music is generally characterized by its gentle, soothing qualities, often featuring a frequency range of 200–1000 Hz and a tempo of 60–80 beats per minute, creating a calming and non-distracting atmosphere. Studies have found that light music can effectively relieve symptoms of depression, especially by improving mood and promoting behavioral activity in rats. For example, playing light music increased exploratory behaviors and significantly improved activity levels in depressed rats [72]. This effect might be related to the activation of brain regions associated with relaxation and pleasure, such as the limbic system [73]. Therefore, light music might have potential as a treatment for mild depression or anxiety-related disorders [74].

### 6.3. Pop Music

Pop music is characterized by its catchy melodies, simple structures, and wide ap-peal. In depression treatment, it typically features a tempo range of 100–130 beats per minute (BPM) and a frequency range of 500–5000 Hz. Studies in depressed rats have shown that playing pop music can regulate their emotional states and significantly improve depressive behaviors [75]. For example, pop music with a bright rhythm reduced the immobility time of rats in the tail-hanging experiment, indicating a stimulating effect [59]. In addition, the emotional appeal of pop music may enhance positive responses in rats by modulating endogenous reward systems, such as the dopamine system [75].

### 6.4. Other Types of Music

In addition to the three main types of music mentioned above, meditation music and nature sounds (e.g., ocean waves, bird songs) have also been explored in depression intervention studies. These types of music, which typically have milder rhythms and are less stimulating, can promote relaxation and mood regulation in mice, which can reduce pain or depressive symptoms [76]. Meditation music, in particular, has shown positive effects in reducing anxiety and promoting mental relaxation. Studies have shown that meditation music can effectively regulate neurotransmitter levels and improve behavioral activity in rats [14].

## 7. Future Challenges and Perspectives

Music therapy, an interdisciplinary field integrating music, medicine, and psychology, highlights the practical applications of music beyond artistic appreciation and aesthetics. It has become an important part of the global medical field, significantly impacting people’s health. In particular, the intervention effects of music therapy on depressed rat models have shown considerable potential. However, challenges and opportunities remain.

(1) The personalization of music therapy is a key direction for future research. As individuals respond differently to music, future studies should explore how personalized music therapy programs can be tailored to a patient’s musical preferences, cultural background, and emotional state, while also considering the biophysical aspects of music such as volume, tempo, timbre, duration of tracks, and frequency range. This personalized treatment may be achievable through precision medicine, which combines genotypic and phenotypic information to optimize treatment outcomes [77,78]. With the development of big data and machine learning technologies, we can expect more accurate, personalized music therapy solutions to emerge.
(2) Addressing Species-Specific Differences and Translational Limitations: Future research should also focus on understanding the species-specific differences in neurobiology and music perception between rodents and humans. This is crucial for translating the findings from rodent models to human applications. Strategies to address these limitations could include developing more sophisticated animal models that better mimic human conditions, as well as conducting parallel studies in both rodents and humans to validate the translational potential of music therapy.
(3) Addressing Sample Size and Long-Term Effects: Future research should address the limitations of small sample sizes and short-term focus by conducting studies with larger cohorts and long-term follow-ups. This will enhance statistical power and provide clearer insights into the lasting effects of music therapy. Additionally, future studies should include appropriate controls, such as non-musical auditory stimuli or silence, to confirm the specificity of music’s effects. Meanwhile, with advances in remote monitoring technologies, it may be easier in future studies to assess the long-term effects of music therapy [79].
(4) A deep understanding of the neurobiological mechanisms of music therapy is essential for improving therapeutic outcomes. Future research should focus on how music affects brain function and neurotransmitter levels, as well as how these changes correlate with improvements in depression. These studies may be conducted using neuroimaging techniques and molecular biological methods [80,81]. For example, music has been found to improve the behavior of depressed mice by regulating the levels of serotonin (5-HT) [47]. With advances in neuroscience, we hope to uncover the specific effects of music therapy on brain structure and function.
(5) Combining music therapy with other therapeutic approaches (e.g., drug therapy, psychotherapy) may improve therapeutic outcomes. Future research is needed to explore the optimal combination and timing of these multimodal interventions to maximize the therapeutic effects. Technological innovations, such as virtual reality (VR) and artificial intelligence (AI), present new possibilities for music therapy. AI can analyze rats’ emotional responses in real time and adjust musical stimuli, while VR can provide an immersive environment for musical experiences.
(6) The cost-effectiveness and acceptability of music therapy make it an important component of public health strategies, particularly in preventing depression and promoting mental health. With the development of Internet technology, remote music therapy has become possible, enabling more rats to receive treatment in experimental settings. The realization of teletherapy will depend on the advancements in web technology and mobile applications, which will make music therapy more convenient and accessible.

## 8. Conclusions

In conclusion, this study provides a comprehensive review and constructive discussion on the progress of music therapy for depression, particularly based on the depressed rat model. Although advancements have been made in this field, the practical application of music therapy as a treatment is still a long way off. This paper highlights the challenges and prospects of music therapy for depression. It is anticipated that this review will pave the way for both basic research and the clinical application of music therapy in the treatment of depression.
